# Long-Term Structural and Functional Myocardial Adaptations in Healthy Living Kidney Donors: A Pilot Study

**DOI:** 10.1371/journal.pone.0142103

**Published:** 2015-11-10

**Authors:** Diego Bellavia, Alessandro Cataliotti, Francesco Clemenza, Cesar Hernandez Baravoglia, Angelo Luca, Marcello Traina, Bruno Gridelli, Tullio Bertani, John C. Burnett, Cesare Scardulla

**Affiliations:** 1 Division of Cardiovascular Diseases, Mediterranean Institute for Transplantation and Advanced Specialized Therapies (ISMETT), Palermo, Italy; 2 Cardiorenal Research Laboratory, Division of Cardiovascular Diseases, Mayo Clinic, Rochester, Minnesota, United States of America; 3 Institute of Clinical Medicine and Institute for Experimental Medical Research, University of Oslo and Oslo University Hospital, Oslo, Norway; 4 Department of Radiology, Mediterranean Institute for Transplantation and Advanced Specialized Therapies (ISMETT), Palermo, Italy; 5 Sport and Exercise Sciences "DISMOT" Research Unit, University of Palermo, Palermo, Italy; 6 Division of Nephrology, Mediterranean Institute for Transplantation and Advanced Specialized Therapies (ISMETT), Palermo, Italy; University Medical Center Utrecht, NETHERLANDS

## Abstract

**Background and Aims:**

Compensatory renal hypertrophy following unilateral nephrectomy (UNX) occurs in the remaining kidney. However, the long-term cardiac adaptive process to UNX remains poorly defined in humans. Our goal was to characterize myocardial structure and function in living kidney donors (LKDs), approximately 12 years after UNX.

**Methods and Results:**

Cardiac function and structure in 15 Italian LKDs, at least 5 years after UNX (median time from donation = 8.4 years) was investigated and compared to those of age and sex matched U.S. citizens healthy controls (n = 15). Standard and speckle tracking echocardiography (STE) was performed in both LKDs and controls. Plasma angiotensin II, aldosterone, atrial natriuretic peptide (ANP), N terminus pro B-type natriuretic peptide (NT-proBNP), cyclic guanylyl monophosphate (cGMP), and amino-terminal peptide of procollagen III (PIIINP) were also collected. Median follow-up was 11.9 years. In LKDs, LV geometry and function by STE were similar to controls, wall thickness and volumes were within normal limits also by CMR. In LKDs, CMR was negative for myocardial fibrosis, but apical rotation and LV torsion obtained by STE were impaired as compared to controls (21.4 ± 7.8 vs 32.7 ± 8.9 degrees, p = 0.04). Serum creatinine and PIIINP levels were increased [1.1 (0.9–1.3) mg/dL, and 5.8 (5.4–7.6)] μg/L, respectively), while urinary cGMP was reduced [270 (250–355) vs 581 (437–698) pmol/mL] in LKDs. No LKD developed cardiovascular or renal events during follow-up.

**Conclusions:**

Long-term kidney donors have no apparent structural myocardial abnormalities as assessed by contrast enhanced CMR. However, myocardial deformation of the apical segments, as well as apical rotation, and LV torsion are reduced. The concomitant increase in circulating PIIINP level is suggestive of fibrosis. Further studies, focused on US and EU patients are warranted to evaluate whether these early functional modifications will progress to a more compromised cardiac function and structure at a later time.

## Introduction

Kidney transplantation as optimal treatment and standard care for patients with end-stage renal disease (ESRD) confers a survival benefit and is cost effective compared to hemodialysis [1]. It is known that living kidney donation causes functional adaptation and hypertrophy of the remaining kidney (a process named compensatory hyperfiltration). Creatinine clearance of the remaining donor kidney increases to 70%–75% of preoperative value after nephrectomy, while the glomerular filtration rate (GFR) is reduced in an average of 26 ml/min/1.73 m^2^ (range 8–50) after donation [2]. Given diminished renal function in conjunction with the presence of a solitary kidney, many donors would be considered to meet criteria for chronic kidney disease (CKD). Several reports suggest favorable long-term outcomes and longer life expectancy for donors compared to the rest of the population, perhaps due to selection of exceptionally healthy persons. However, it is also evident that development of hypertension, proteinuria, or acute cardiovascular events, increases significantly the risk of developing ESRD in donors [3–5]. Moreover, a recent report in a model of mild renal insufficiency produced by unilateral nephrectomy (UNX) suggests that removal of a single kidney induces pathologic myocardial accumulation of extracellular matrix components [6], a fibrotic process that has been increasingly implicated as a major cause of cardiovascular morbidity and mortality in humans. The availability of biochemical markers of collagen biosynthesis, and extremely sensitive imaging modalities, offers the invaluable opportunity to detect significant myocardial abnormalities in the pre-clinical stage [7].

To date the long-term cardiac adaptation to UNX remains poorly defined in living kidney donors (LKDs). Thus, our aim was to determine subclinical myocardial fibrosis and concomitant left ventricular (LV) impairment in a small but highly homogeneous sample of caucasian, healthy, long-term donors (LKDs), with an integrated approach involving assessment of established cardiac biomarkers including plasma procollagen type III N-terminal propeptide (PIIINP), and state of the art myocardial imaging technologies including gadolinium delayed enhancement cardiac magnetic resonance (CMR), and 2D speckle strain echocardiography. We hypothesized that, by using these high resolution imaging modalities in combination with established and novel humoral markers of function and remodeling, early preclinical signs of impaired function and fibrosis can be detected in otherwise clinically normal LKDs.

## Methods

### Study Population

This cross-sectional study was approved by the Institutional Review Boards of both the Mediterranean Institute for Transplantation and Advanced Specialized Therapies (ISMETT), Palermo, Italy, and the Mayo Clinic, Rochester, MN, USA.

Subjects who donated kidneys at ISMETT between January 2000 and December 2004 were considered for recruitment. These group of donors was called back to ISMETT and underwent a new clinical, laboratory and imaging work-up. At the same time a group of age- and sex-matched healthy subjects was identified at the Mayo Clinic. Finally, 15 LKDs and 15 age and sex comparable controls were recruited from June 2010 through June 2011. Follow-up ended on April 30^th^ 2014. For LKDs the inclusion criteria was UNX performed 5 years before enrollment date. Healthy controls were selected from a pool of subjects extensively studied to exclude any morbidity, as well as known cardiovascular risk factor: smokers, subjects with BMI ≥ 30, patients with dyslipidemia, diabetes, or with history of pre-hypertension(i.e. blood pressure > 120/80 mmHg) were excluded. For donors, the exclusion criteria were presence of diabetes (either type I or II, n = 0), stage II hypertension (defined as blood pressure ≥ 160/95 mmHg, n = 1), impaired renal function at the baseline (≥ stage III chronic kidney disease, n = 0), or COPD (n = 0). Finally, patients with clinical evidence of myocardial ischemia (n = 0), those referred to coronary revascularization (n = 0), significant valvular heart disease (n = 1), chronic atrial fibrillation (n = 0), permanent pacemaker (n = 0), or left bundle branch block (n = 0), were also excluded. According to the IRB committees at both recruiting centers (Mayo and ISMETT), each subject (either patient or control) eventually enrolled, provided written informed consent to participate to the study. At the end of follow-up (April 30^th^ 2014) both controls and donors were contacted by telephone to ascertain primary end-point of all-causes death, ESRD, or a major cardiovascular event, defined as myocardial infarction, stroke, coronary angioplasty, coronary bypass surgery, carotid endarterectomy, abdominal aortic aneurysm surgery or peripheral vascular bypass surgery.

### Neurohumoral Profile and Cardiorenal Biomarkers

In patients and controls, complete metabolic panel and liver function tests, as well as serum levels of aldosterone, plasma renin activity (PRA), atrial natriuretic peptide (ANP), N-terminal pro-natriuretic peptide B (NT-proBNP), cyclic guanylyl monophosphate (cGMP), were measured at the time of recruitment. Urinary level of cGMP was also measured. Plasma PIIINP, cystatin C, and angiotensin II was also collected in LKDs. GFR was calculated using the CKD-EPI Study equation [8].

PIIINP was measured using a radioimmunoassay kit (UniQ PIIINP RIA; Orion Diagnostics, Fountain Hills, Arizona)[9]. Plasma NT-proBNP levels were measured with electrochemiluminescence sandwich immunoassay (ECLIA; Roche Diagnostics; Indianapolis, Indiana) using the Elecsys System 2010. ANP, and cGMP, PRA, angiotensin II, and aldosterone were determined by standard radioimmunoassay techniques as previously described [10]. Technicians were blinded to the identity of the patients.

### Echocardiography

All ultrasound examinations were performed with a commercially available echocardiographic instrument (Vivid 7 System, Vingmed, General Electric Healthcare; Milwaukee, Wisconsin). A standard two-dimensional and flow Doppler echocardiogram was performed at enrollment. Left ventricular (LV) end-diastolic dimensions (LVEDd) and LV end-systolic dimensions (LVESd), as well as LV wall thickness, and left atrial volume were measured; left atrial volume was indexed to body surface area. LV ejection fraction was computed as [(LVEDd2—LVESd2)/LVEDd2], as reported elsewhere [11]. Assessment of diastolic function was performed by trans-mitral early (E wave velocity) and late (A wave velocity) Doppler flow waves, E/A ratio and E deceleration time, and measuring the early diastolic velocity at the medial mitral annulus (E’) by pulsed wave tissue Doppler. E/E’ ratio was used as a parameter of LV end-diastolic filling pressure, as previously shown [12]. In addition, a full speckle myocardial imaging study, focused on systolic strain (sS) as well as systolic (s) and diastolic (d) strain rate (SR) assessment along longitudinal, radial, and circumferential LV axes was completed, using a model of 18 LV segments. The 18-segments model was preferred to the standard 17-segments model defined by the American Society of Echocardiography since GE Vivid machines consider 6 LV apical segments instead of 5 [13]. Basal and apical rotation was assessed, and LV torsion was computed as the difference between apical and basal rotation [14]. Torsion and detorsion velocities were collected as well. For longitudinal speckle myocardial imaging, analysis was performed considering all 18 LV segments individually and also combining them in clusters according to LV level (average of the 6 basal, the 6 middle and 6 apical segments), and LV wall (mean of the basal, middle and apical segment for each of the 6 LV walls: anterolateral, inferoseptal, inferolateral (i.e. posterior), anteroseptal, inferior and anterior).

### Ecg-Gated Cardiac Magnetic Resonance Imaging

ECG-gated cardiac magnetic resonance (CMR) was performed in each patient within one month from the ultrasound evaluation and blood and urine collection. Testing was performed with a 1.5-Tesla system (Twin speed EXCITE, GE Healthcare, Waukesha, WI) within six months from recruitment date. Images were acquired during repeated end-inspiratory breath holds. Sagittal fast gradient echo scout images were used to prescribe subsequent steady-state free precession (SSFP) cine-sequences. Long axis cine SSFP images were obtained, including a 4-chamber, 2-chamber and 3-chamber cine SSFP. From the 4-chamber images, short axis cine SSFP images were prescribed. LV function, volumes, and ejection fraction was calculated using commercial software (GE, MASS Analysis 6+) by summation of discs. A multi-inversion time inversion recovery sequence was performed in a mid-LV short-axis slice 5–10 minutes after gadolinium to provide the optimal inversion time for myocardial nulling. Delayed enhancement images covering the entire LV in multiple short-axis and at least three long-axis views were obtained 5–10 minutes after a total intravenous bolus of 0.2 mmol/kg gadolinium-DTPA (Magnevist ^®^) with inversion recovery prepared fast gradient echo sequences [15]. The inversion time was individually adjusted to optimally null the myocardial signal. Analysis was performed of both myocardial and blood pool nulling patterns as well as of the presence and pattern of myocardial delayed enhancement.

### Statistical Analysis

Statistical analyses were performed with a commercially available software program (STATA 13.1, MP version, statacorp. Ltd, College Station, Texas). Comparisons between patients and controls were made by Wilcoxon-Mann-Whitney test or Fisher exact test, as appropriate. Finally, in order to prevent false positive results due to significantly different systolic blood pressure (SBP) between donors and controls, we have repeated comparisons stratifying groups by the median SBP (120 mmHg): Group I included subjects (either controls or donors) with SBP ≤ 120 mmHg, while Group II included subjects with SBP > 120 mmHg.

To examine intra-observer variability (repeatability), a sample of 10 echocardiographic examinations were randomly selected for masked review by the same investigator. To examine inter-observer variability a co-investigator blinded to the clinical information and to the results of the first investigator examined 10 randomly selected echocardiographic exams. Intra-class correlation coefficients (ICCs) for the same observer and different observers were calculated using previously described formulae [16] for the global mean of longitudinal sS, dSR-E, and for LV torsion.

A confidence interval of 95% was chosen as significant, with α = 0.05. Data were expressed as mean ± standard deviation (SD), median (inter-quartile range (IQR, 25^th^ to 75^th^ percentile)), or count (percentage).

## Results

### Participant Characteristics and Biomarkers

By study design, all patients were considered for enrollment at least 5 years after UNX; however, actual median time from donation was 8.4 years, while median follow-up was 11.9 years. Of note, no enrolled subject (either non-donor or LKD) developed primary endpoint during follow-up. Demographic, clinical and biochemical characteristics of LKD and controls are shown in Table 1: age, gender and height/weight were similar in both groups. No enrolled subject (either patient or control) was a smoker, and there were no cases of dyslipidemia, diabetes or chronic obstructive pulmonary disease (COPD). One LKD had a diagnosis of grade I hypertension which was controlled by diet and physical exercise. No subject enrolled was on medication. The serum creatinine level was higher in LKD as compared to controls while BUN and GFR were similar in the 2 groups (Table 1). Serum electrolytes, as well as liver function tests were not different in donors compared to controls. Regarding advanced biomarkers, PRA, plasma ANP and both plasma and urinary cGMP were significantly lower in LKD compared to controls. Plasma NT-proBNP and aldosterone trended to be lower in LKDs as compared to controls, although this difference was not significant. Serum levels of cystatin C, and angiotensin II were also in the normal range (Table 1) [17]. Of note, PIIINP levels were elevated in all of the donors, according to the reported range of normality [18].

**Table 1:** (Definitive). Biometrics, Clinical characteristics and Biomarkers. Clinical variables, metabolic panel, liver function tests, and cardiovascular biomarkers in living kidney donors and controls. Descriptions are with mean ± standard deviation (SD), median (inter-quartile range) or count (percent).

	Controls	Kidney Donors	P-Value
Mean ± SD or Median (IQR)	(N = 15)	(N = 15)	
Age (years)	60 ± 6	57 ± 6	0.19
Gender (Females(%))	8 (53)	8 (53)	0.64
Height (mt)	1.71 ± 0.1	1.67 ± 0.1	0.25
Weight (Kg)	81 ± 21	73 ± 15	0.35
Body Mass Index	27 ± 4	26 ± 4	0.39
Systolic Blood Pressure (mmHg)	102 ± 19	123 ± 8	0.45
Diastolic Blood Pressure (mmHg)	71 ± 10	74 ± 8	0.19
Heart Rate (bpm)	67 ± 12	71 ± 10	0.40
Blood Urea Nitrogen (mg/dL)	31 (23–34)	39 (31–47)	0.11
Creatinine (gr/dL)	0.8 (0.7–0.9)	1.1 (0.9–1.3)	0.01
eGFR by CKD-EPI equation (ml/min/1.73m^2^)	87 (69–99)	75 (60–82)	0.12
ANP (pg/mL)	17.15 (7.3–29.6)	2.5 (2–3.1)	0.007
NTproBNP (pg/mL)	46.7 (32.2–58.8)	32 (10–78)	0.13
Aldosterone (ng/dL)	12 (5.9–17.7)	6.7 (4–14.2)	0.14
PRA (ng/mL/hr)	1.35 (0.7–2)	0.19 (0.2–0.2)	0.001
cGMP_Plasma (pmol/mL)	1.75 (1.4–2.4)	1.3 (1.1–1.3)	0.02
cGMP_Urine (pmol/mL)	581 (436.6–698)	270.1 (250.5–354.7)	0.004
Angiotensin II (pg/mL)	(0.8–16)	1.4 (0.5–3)	N/A
PIIINP (μg/L)	(2.3–6.4)*	5.8 (5.4–7.6)	N/A
Cystatin C (mg/L)	(0.57–1.12)*	0.97 (0.92–1.02)	N/A

GFR = Glomerular filtration rate.

* = normal range limits accordig to the literature

### Standard and Strain Echocardiography

LV geometry (as assessed by LV wall thickness and diameters) was normal and not different in the 2 groups (Table 2). Left atrial volume was higher in controls as compared to LKDs. LV ejection fraction (EF) was similar in the 2 groups, while stroke volume, cardiac output and cardiac index were lower in LKDs as compared to controls (although they were within normal range for both groups). Diastolic function as assessed by trans-mitral flow Doppler was not different in LKDs or control subjects, LV end-diastolic filling pressures were also similar. The diastolic E’ velocity at the medial mitral annulus, measured from pulsed-wave tissue Doppler imaging, was normal and similar in LKDs and controls.

**Table 2:** Standard ECHO and Cardiac MRI. Two-dimensional and standard Doppler echocardiographic variables in living kidney donors and controls. Contrast-Enhanced Cardiac Magnetic Resonance data obtained on living kidney donors are reported as well.

	Controls	Kidney Donors	P-Value
Mean ± SD	(N = 15)	(N = 15)	
Standard ECHO			
LV wall Thickness (mm)	10 (9.5–10)	9 (8.5–10)	0.103
LV End-Diastolic Diameter (mm)	46.7 ± 6	45.6 ± 6	0.65
LV End-Systolic Diameter (mm)	29.4 ± 4.5	31.6 ± 5	0.39
EF (%)	65 ± 3	62 ± 6	0.236
Stroke Volume (cc)	94 ± 26	71 ± 13	0.009
Cardiac Output (L/min)	6.2 ± 1.4	5.01 ± 0.8	0.027
Cardiac Index (L/m2/min)	3.21 ± 0.5	2.75 ± 0.4	0.016
Left Atrial Volume Indexed (mL/m2)	31 ± 8.6	24 ± 5	0.014
E wave velocity (m/sec)	0.75 ± 0.2	0.67 ± 0.1	0.165
A wave velocity (m/sec)	0.64 ± 0.2	0.65 ± 0.1	0.979
E/A ratio	1.24 ± 0.4	1.04 ± 0.3	0.268
E wave deceleration time (msec)	195 ± 30	185 ± 23.8	0.328
E' velocity (m/sec)	0.09 ± 0.01	0.09 ± 0.01	0.875
E/E' ratio	8.6 ± 2.7	7.8 ± 2.5	0.411
Cardiac Magnetic Resonance			
LV Anteroseptal Wall thickness (mm)	N/A	9.98 ± 1.4	
LV Posterior Wall thickness (mm)	N/A	8.12 ± 0.8	
LV End Diastolic Volume Indexed (mL/m2)	N/A	57.45 ± 9	
LV End Systolic Volume Indexed (mL/m2)	N/A	19.09 ± 5.9	
LV EF (%)	N/A	66 ± 5	
LV Stroke Volume (cc)	N/A	74 ± 14	

ECHO = echocardiography; MRI = Magnetic Resonance Imaging

### Longitudinal Axis

**Fig 1:**
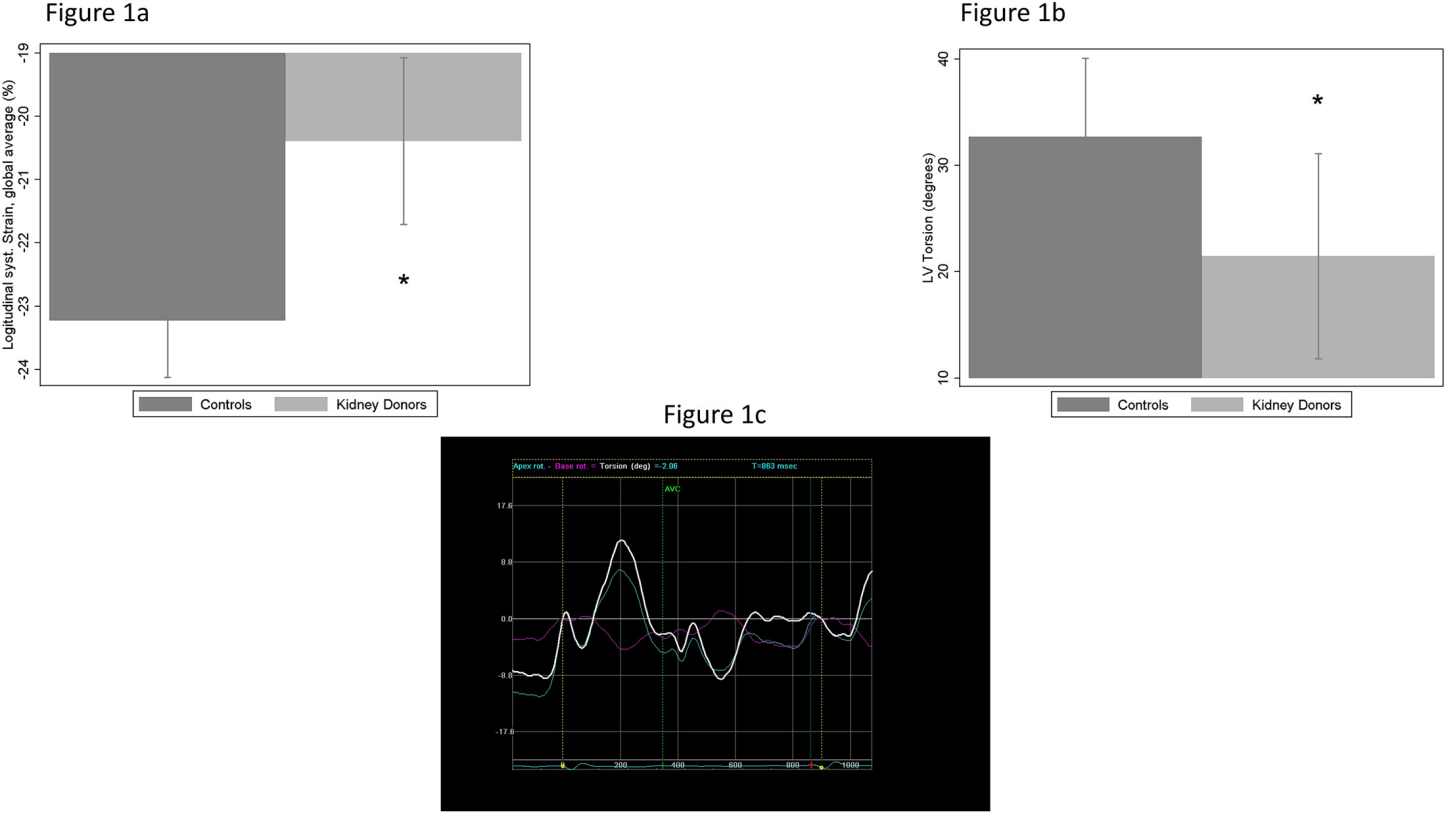
Bar graph showing mean (± SD) of different speckle myocardial imaging modalities in kidney donors (n = 15) and controls (n = 15). (a) Longitudinal systolic strain, global average of 18 LV segments, in the 2 groups under study; star = p < 0.05 for the comparison between kidney donors and controls (Mann-Withney Test). (b) Left ventricular torsion in the 2 groups under study; star = p < 0.05 for the comparison between kidney donors and controls (Mann-Withney Test). (c) Example of a typical curve showing LV torsion rate (degrees/sec, positive wave) and de-torsion rate (early: first negative wave, late: second negative wave) obtained on a healthy subject with the GE ECHOPAC software.

Longitudinal sS was lower in LKDs as compared to controls, when considering the mean of the basal, and of the apical segments, as well as clusters of segments by the LV walls (Table 3). The mean of the middle segments was only marginally significant though. Although the global average sS of the 18 LV segments was reduced in kidney donors (Fig 1a), sS measurements were within normal range in both groups. Longitudinal sSR values, considered as either global average of all 18 LV segments or as the sSR mean of basal, middle, or apical segments, were normal and similar in both groups. Diastolic longitudinal sSR values assessed at early diastole (dSR-E) were reduced in LKDs as compared to controls, considering apical segments, or the global dSR-E average of 18 LV segments.

**Table 3:** Cardiac Mechanics and Rotation/Torsion, by ECHO Strain. Speckle myocardial Imaging data over longitudinal, radial and circumferential LV axes in kidney donors and controls. Basal/Apical Rotation and Torsion of the LV in the 2 groups are reported as well.

	Controls	Kidney Donors	P-Value
Mean ± SD	(N = 15)	(N = 15)	
Longitudinal systolic Strain (%)			
Mean of the Basal Segments	-22.97 ± 1.8	-20.46 ± 2.8	0.033
Mean of the Middle Segments	-23.07 ± 1.6	-21.44 ± 1.7	0.061
Mean of the Apical Segments	-23.57 ± 2.2	-19.01 ± 1.9	0.001
Global Average	-23.23 ± 1.6	-20.39 ± 1.6	0.02
Longitudinal systolic Strain Rate (%/sec)			
Mean of the basal segments	-1.55 ± 0.3	-1.44 ± 0.1	0.651
Mean of the middle segments	-1.39 ± 0.2	-1.28 ± 0.1	0.366
Mean of the apical segments	-1.47 ± 0.3	-1.3 ± 0.2	0.175
Global Average	-1.47 ± 0.2	-1.34 ± 0.1	0.197
Longitudinal diastolic Strain Rate (early diastole, %/sec)			
Mean of the basal segments	1.95 ± 0.3	1.7 ± 0.3	0.061
Mean of the middle segments	1.7 ± 0.3	1.5 ± 0.3	0.333
Mean of the apical segments	2 ± 0.6	1.3 ± 0.2	0.004
Global Average	1.89 ± 0.3	1.5 ± 0.2	0.008
Radial/Circumferential Strain (%) and Strain Rate (%/sec)			
Radial systolic Strain Rate	1.99 ± 0.5	2.58 ± 1	0.04
Radial diastolic Strain Rate	-2.14 ± 0.8	-3.03 ± 0.8	0.024
Radia systolic Strain	48.96 ± 13.5	55.13 ± 21.3	0.453
Circumf. Systolic Strain Rate	-1.94 ± 0.6	-2.04 ± 0.3	0.359
Circumf. Diastolic Strain Rate	2.46 ± 0.5	1.85 ± 0.3	0.006
Circumf. Systolic Strain	-25.52 ± 3.9	-17.96 ± 3.4	0.001
Rotation and Torsion			
Rotation, Basal segments (degrees)	-8.5 ± 2.8	-9.5 ± 3.4	0.477
Rotation, Apical Segments (degrees)	26.6 ± 12.2	13.1 ± 3.4	0.03
Left ventricular Torsion (degrees)	32.7 ± 8.9	21.4 ± 7.8	0.04
Torsion Rate (degrees/sec)	193.34 ± 55.3	167.24 ± 61.8	0.245
DeTorsion Rate (degrees/sec)	-194.45 ± 44.7	-131.17 ± 45.5	0.02

### Radial and Circumferential Axes

Radial sSR and dSR-E were higher in donors as compared to controls, while radial sS was similar between LKDs and controls. Circumferential sSR was similar in the 2 groups as well, while circumferential dSR-E and sS values were reduced in donors as compared to controls.

### Rotation and Torsion

LV apical rotation was significantly reduced in the LKDs, while rotation of the basal segments was comparable between the groups. As result of apical rotation reduction, LV global torsion was significantly reduced in LKDs when compared to controls (Fig 1b). Finally, LV torsion rate was similar between groups, while LV detorsion rate was lower in LKDs.

The intra-class correlation coefficient (ICC) for intra-reader reproducibility was greater for LV torsion (0.86 [95% CI 0.50, 0.96]) and longitudinal sS (0.83 [0.45, 0.95]) than for longitudinal dSR-E (0.78 [0.24, 0.90]). The ICC for inter-reader reproducibility was higher for longitudinal sS (0.87 [0.56, 0.97]) and LV torsion (0.77 [0.20 0.89]) than for longitudinal dSR-E (0.72 [0.17, 0.89]).

#### CMR

**Fig 2:**
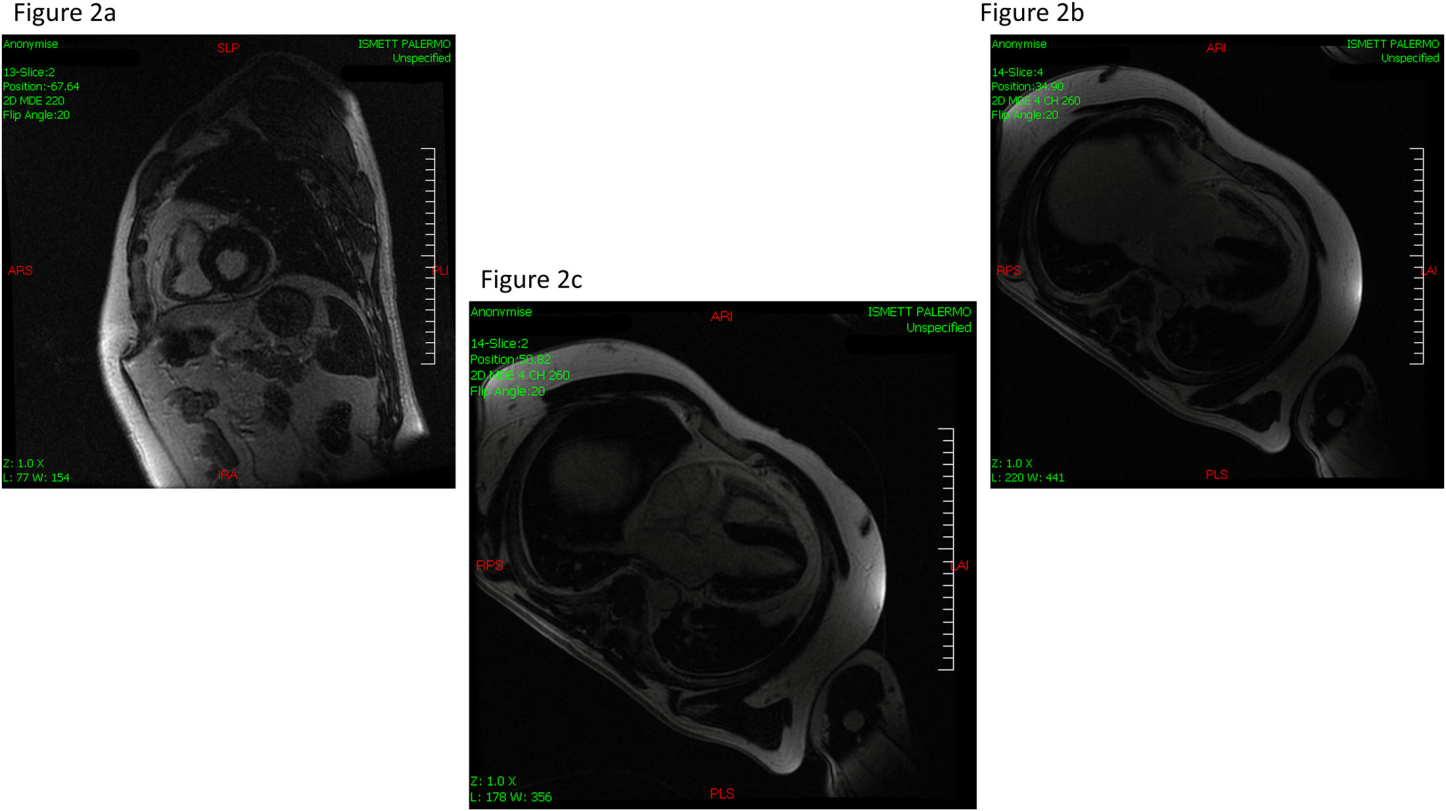
Late Gadolinium Enhanced Cardiac Magnetic Resonance in the only kidney donor positive for myocardial fibrosis. Short axis view at the middle level, delayed enhancement suggestive of patchy fibrosis at the inferior wall. (a) Four chambers view, delayed enhancement suggestive of patchy fibrosis at the basal lateral segment. (b) Four chambers view, delayed enhancement suggestive of patchy fibrosis at the basal lateral segment.

LV wall thickness, LV volumes and EF, evaluated by CMR, were normal in all LKDs, and consistent with echocardiographic measures. Delayed enhancement, as assessed by CMR with gadolinium, was present in the one patient with grade I hypertension. In this single case, delayed enhancement was localized to the basal segment of the infero-lateral wall, and to the middle segment of the lateral wall (Fig 2a, 2b and 2c).

#### Comparisons of Groups by Median Systolic Blood Pressure

The analyses outlined above have been repeated stratifying groups by SBP (Group I, SBP ≤ 120 mmHg, N = 17 vs Group II, SBP > 120 mmHg, N = 13) irrespective of control or donor status. According to this stratification criterion, age and radial diastolic strain rate at early diastole were the only values to be different between Group I and Group II (p = 0.01, and p = 0.02, respectively, see S1 Table, online material). In particular no biochemical or echocardiographic parameter (including strain imaging values) was different between groups defined by SBP.

## Discussion

The present study was designed to investigate the long-term cardiac adaptation to UNX in LKDs. To achieve this goal, we enrolled 15 highly homogeneous “supernormal” renal donors, at least five years post UNX (real median time from donation = 8.4 years), and determined cardiac function and structure by combining state of the art in-depth imaging modalities, including ECG-gated CMR and echo strain analysis, with levels of established and emerging humoral markers. We further compared these findings to those obtained in age and gender matched healthy control subjects, free of known cardiovascular risk factors. The findings of the current investigation suggest that in LKDs there is evidence of mild, pre-clinical myocardial dysfunction, affecting LV deformation both in systole and in diastole, localized at the apex, and associated with abnormal torsion/detorsion of the LV. However, after ~ 8 years post UNX, these early anomalies were not accompanied by signs of myocardial fibrosis/remodeling, as determined by contrast enhanced CMR. Nevertheless, an elevated level of PIIINP was observed that suggests development of fibrosis possible related to extra-cardiac fibrosis or occult cardiac fibrosis.

Cardiovascular disease is a leading cause of morbidity and mortality for individuals with low GFR in the general population [19], and is the main cause of death among living kidney donors, accounting for approximately 30–40% of all deaths [1, 4]. Classic studies have established that mortality and CV risk rise with even mild CKD and increase with the reduction of renal function [19]. However, more recent reports are inconsistent in that regard: a retrospective analysis performed in Ontario, Canada, comparing LKDs to carefully matched non-donors showed that Donors were at lower risk of death or major cardiovascular events than were nondonors [20], while in a recent study conducted in Norway, a follow-up period of 15 years demonstrated a higher risk of cardiovascular death among LKDs than among healthy non donors [4]. Preclinical investigation also has suggested increased myocardial fibrosis post UNX [6]. This is important because a significant increase in myocardial collagen content is always present in end-stage heart failure as well as in myocardial damage [6], which may be associated with worsening ventricular systolic function, abnormal cardiac remodeling, increased ventricular stiffness, and greater risk of cardiovascular morbidity and mortality.

In spite of this evidence, several studies showed that kidney donors do not incur shortened survival: Fehrman-Ekholm et al.[1] found that kidney donors live longer than a general Scandinavian population, and an extensive single-center study, of nearly 3700 kidney donors spanning four decades, reported that the 20-year survival was 93.5% for renal donors compared to 89.5% of age-matched controls [21]. Although these results are reassuring, they might also be expected given the rigorous screening and selection of donors to ensure good health before donation. To overcome this selection bias, one study compared long-term survival among 80,347 living kidney donors in the USA with that of an age-matched and comorbidity-matched cohort of 9,364 participants of NHANES III who did not have comorbidities that preclude donation: mortality during the decade after kidney donation was lower among living kidney donors than among the healthy matched nondonors [22]. In contrast, Mjoen et al. from the University of Oslo have recently reported that renal donation is associated with increased risk for death, ESRD and cardiovascular disease [4]. It is noteworthy though that the Norwegian study has several limitations, including different accrual periods and differences in baseline characteristics between donors and nondonors (for example, donors were older than non-donors at baseline (46 years versus 38 years); finally, concerns have also been raised about whether the control group is representative of the Norwegian population, as the survey was conducted in the rural county of Nord-Trøndelag, where life expectancy exceeds the national average. However, it is noteworthy that median follow-up time was signficantly lower in the NHANES III study (median (interquartile range) follow-up = 6.3 (3.2–9.8) years) as compared to the study by Mjoen and co-workers (median (interquartile rage) follow-up = 15.1 (1.5–43.9) years); it is therefore reasonable to assume that an adequately longer follow-up is needed to uncover differences in mortality among LKDs. It is also interesting to note that a more recently published post-hoc analysis of the NHANES III cohort, implementing a better matching algorithm between LKD and healthy controls has demonstrated a slight but significant increase in absolute risk of developing ESRD among donors, in a median follow-up time of 7.3 years(5).

We studied the possible long-term cardiac adaptation to UNX in a small sample of highly selected long standing Italian LKDs, using thorough imaging investigation with state of the art contrast-enhanced CMR and echocardiography in a cross-sectional study design. Here, similar to what has been reported in these previous studies, during a median follow-up period of almost 12 years, none of our patients died, or developed CV events or ESRD. Only one patient presented stage I hypertension at the time of evaluation, which was controlled with diet and exercise, without clinically evident signs of progression. LV geometry and function of LKDs, as assessed by standard echocardiography and CMR, were normal and comparable to healthy controls. Importantly, CMR provides comprehensive assessment of myocardial anatomy and function, with unmatched levels of accuracy and reproducibility. The use of gadolinium contrast agent has further enhanced the accuracy and precision of this approach to analyze myocardial tissue composition, especially myocardial fibrosis content. In order to detect any myocardial pathologic process, we purposely enrolled patients who donated several years before evaluation. After an average time of 8.4 years post UNX, subtle myocardial fibrosis was detected only in the donor with stage I hypertension, but in none of the other donors. The lack of cardiac fibrotic deposition was also consistent with the normal LV volumes and performance (both systolic and diastolic) observed with standard echocardiography, and with the event free follow-up.

It is perhaps more difficult to explain the significant reduction in myocardial deformation as assessed by speckle myocardial imaging, as well as the abnormal LV twisting parameters we observed in LKDs. First, global reduction in myocardial deformation does not involve radially oriented myofibers, and this is consistent with the normal LV ejection fraction observed in donors. Second, although longitudinal sS and dSR-E are lower in LKDs compared to our controls, the values are well inside the accepted range of normality [23]. This is specifically true for basal and middle segments, and the discrepancy observed between LKDs and controls is consistent with the same difference observed in stroke volume, cardiac output, and cardiac index between the 2 groups. If this was the only observation, one could argue that difference can be explained simply by different contingent volemic conditions and, therefore, by a different pre- or after-load status between patients and controls. However, deformation of the longitudinally oriented fibers of the apical segments in systole, and in early diastole, was clearly on the lower limit of normality on average, and abnormally low for some of the patients. At the same time global deformation and deformation rate along the circumferentially oriented myocardial fibers was reduced in LKDs, and parameters such as detorsion rate were abnormally low in donors as compared to controls. Finally, LKDs had an abnormally low rotation of apical segments, explaining the reduced global LV torsion in this group. LV torsion is considered an extremely sensitive technique to detect initial, subtle myocardial dysfunction [24], comparable to longitudinal sS in terms of sensitivity and diagnostic accuracy [14]. In summary, kidney donors had an abnormal reduction in deformation parameters, involving longitudinally oriented fibers and particularly localized at the apical segments, thus affecting LV rotation and torsion. Although this abnormality is subclinical, does not affect clinical outcome in the following years, and is inconsistent with contrast enhanced CMR, a discrepancy between CMR and functional assessment by speckle myocardial imaging is not uncommon [25], and possible clinical consequences in the longer term cannot be excluded. These findings could also be explained by a toxic state related to UNX that has affected myocardial fiber contractility. Finally, interesting data was derived from the biochemical assessment of these patients. First of all, LKDs have no signs of the neuro-hormonal adaptations observed in heart failure. In particular, and consistent with imaging and clinical data, renin-angiotensin-aldosterone levels as well as the natriuretic peptides were not elevated in LKDs, even several years after donation. Considering the pro-fibrotic role of angiotensin II and aldosterone, this observation could also explain the lack of myocardial fibrosis identified by contrast enhanced CMR. Of note, the cardiac biomarkers ANP and cGMP were significantly lower in the LKDs when compared to our control group. This may be due to a selection bias of our control group. Indeed, kidney donors go through rigorous clinical evaluation to confirm good health before UNX, and are usually healthier than general population [1]. Consistently, the evidence that left atrial volume was mildly enlarged in our control group, suggests that elevated LV diastolic filling pressure was responsible for this slightly elevated level of ANP and urinary cGMP (i.e. its second messenger) in our control subjects. It should be noted, however, that NT-proBNP, which is currently considered among the most prominent humoral predictors of cardiovascular events and death in the general population, was similar between the two groups, thus suggesting that the reduced level of ANP may not necessarily translate to an improved survival in LKDs.

In order to definitely ascertain fibrogenesis status in our group of LKDs, we also measured serum level of PIIINP, an extension peptide of type III procollagen that is cleaved during conversion of type III procollagen to collagen. This peptide is then eliminated by the liver (mainly) and by the kidney (secondarily). PIIINP is increased in a number of conditions where increased fibrogenesis, or turnover of connective tissue occurs, and is therefore employed to monitor ventricular remodeling in hypertension, congestive heart failure, and non-ischemic cardiomyopathies [26]. In our group of LKDs, PIIINP was abnormally high, reaching levels that have been previously reported only in advanced stages of dilatative cardiomyopathy or in patients with overt heart failure, where PIIINP level > 4.7 μg/L was an independent predictor of cardiovascular death or hospitalization [27]. Since median PIIINP level in our group of LKDs was 5.8 μg/L, we would have expected higher mortality or morbidity rates during our 12 year follow-up period. This result was even more surprising in light of the above-mentioned negative CMR and biochemical findings, and brought us to the conclusion that, since long-standing kidney donors do not have myocardial fibrosis detectable by CMR, the observed increase in PIIINP level maybe primarily due to extra-cardiac causes with no predictor role for cardiovascular events. Considering that our patients had normal liver function tests and almost normal renal function, the organ with the highest probability of increased fibrotic process would be the kidney, either because of a decreased renal excretion of PIIINP or because of an increased collagen type III production. This hypothesis is speculative since the present study was not designed to assess renal fibrosis. Nevertheless, experimental data have shown that renal damage increases the release of a collagen synthesis stimulating factor [28]. Although several reports have documented the correlation between plasma or urinary PIIINP levels and GFR as well as grade of renal interstitial fibrosis/glomerular sclerosis in patients with acute kidney injury, progressive stages of CKD, and renal transplant recipients [29], it is perhaps not unreasonable to conclude that PIIINP, when unrelated to overt cardiac fibrosis, is not a significant predictor of outcomes. It is noteworthy that a recent work by Dellegrottaglie et al. [30] demonstrated a relationship between indices of arterial stiffness (i.e. pulsed wave velocity) and PIIINP levels, suggesting that other, yet not completely understood, mechanisms can be associated with the observed PIIINP elevation.

### Limitations

The main limitation of the present work is the small sample size. Virtually all studies of long-term outcomes in donors are retrospective (usually surveys on health administrative data), many with large losses to follow-up. Our study is cross-sectional, and although it enrolled a small number of subjects, study population was highly homogeneous, has been studied with the most recent cardiovascular imaging techniques, and no subject was lost to follow-up. Moreover, the observed statistically significant differences between donors and controls suggest adequate power of the study, and low risk of type II error. Of note, we have compared different populations of patients (italians vs US citizens), and although there was no difference in demographic or biometric characteristics, more subtle differences, related to genetic background, culture, and social habits, cannot be excluded: for example, according to the world health organization (WHO) data, italians have on average higher life expectancy as compared to US citizens [31]; consistently, cardiovascular diseases related morbidity and mortality (including hypertension) is higher in United States of America as compared to the European Union countries [32, 33]. Moreover, we have measured several standard as well as advanced cardio-renal biohumoral markers. although we have reported few statistically significant differences between donors and non-donors, the “multiple-comparisons” issue is a real concern in this preliminary report, so that interpreting clinical relevance of such highly variable markers (as demonstrated by the great IQR observed) should be done with caution, and definitely confirmed in bigger populations with longer follow-up.

Understandably, living donors are very carefully screened, and so are inherently healthier at baseline than the general population [21]. Consequently, identification of control group need to be selective as well: In order to create comparable groups, we have purposedly enrolled age-, sex-, and BMI-matched healthy subjects with no known cardiovascular risk factors, employing a strategy similar to that used by other groups [34]. In spite of our effort, controls had higher levels of ANP, cGMP and PRA as compared to donors; however, lack of structural or functional cardiac abnormalities according to 2-D, Doppler, and strain echocardiography in controls, supports the hypothesis that change in bio-humoral markers is due to contingent situations influencing volemic status and LV filling pressures, and not to a significant cardiovascular abnormality. Unfortunately, it was not possible to enroll healthy controls and donors at the same time, so that there was an inconsistency in study design: while controls were healthy at the recruitment time, donors were healthy at the time of donation. How this significant time diference influenced observed findings is unpredictable, although is reassuring that most living kidney donors (all but one) were still in good health at the end of follow-up.

### Conclusions

To our knowledge this is the first investigation to have enrolled and extensively assessed myocardial structure and LV function, using the most accurate and sensitive imaging techniques available today (i.e. CMR and thorough echocardiographic examination), in a homogeneous group of clinically healthy LKDs who donated, on average, 8 years before enrollment. Our findings suggest that LKDs, even several years after UNX, do not develop myocardial fibrosis or abnormalities in LV geometry or function. Risk of cardiovascular or renal events is not increased in a time span reaching 12 years, with normal level of a widely accepted surrogate marker, NT-proBNP. In spite of these reassuring observations, our donors exhibited a mild reduction in systolic as well as diastolic myocardial deformation parameters along the longitudinally oriented myocardial fibers, particularly evident at the apical level, and specifically affecting rotation and torsion of the LV. These alterations have been associated with an increased risk of worsening cardiac function in patients with Non-Compaction cardiomyopathy [14]. Signs of increased extra-cardiac fibrogenesis, possibly of renal origin, were also observed. Nevertheless, the clinical significance of both myocardial strain abnormalities and plasma PIIINP elevation is yet to be defined in LKDs, and further studies, employing quantitative techniques of subtle myocardial fibrosis detection (i.e. T1 Mapping) are warranted.

## Supporting Information

S1 Table “Comparison of Groups by Systolic Blood Pressure (SBP)”. Clinical, laboratory and echocardiographic measurements in recruited subjects (patients and controls), grouped by systolic blood pressure values. Descriptions are with mean ± standard deviation (SD), median (inter-quartile range) or count (percent).
(DOC)
