# Supplementary material for: Long-Term Structural and Functional Myocardial Adaptations in Healthy Living Kidney Donors: A Pilot Study
Source: PLoS One. 2015 Nov 10;10(11):e0142103. doi: 10.1371/journal.pone.0142103 (PMC4640880; doi:10.1371/journal.pone.0142103)
Supplement: S1 Table — Clinical, laboratory and echocardiographic measurements in recruited subjects (patients and controls), grouped by systolic blood pressure values. Descriptions are with mean ± standard deviation (SD), median (inter-quartile range) or count (percent). (DOC) [file pone.0142103.s001.doc]

| **S1 Table: Comparison of Groups by Systolic Blood Pressure (SBP)** | | |  |
| --- | --- | --- | --- |
| Variable | Group I (SBP ≤ 120 mmHg) | Group II (SBP > 120 mmHg) | p-value |
| mean ± SD, or median (IQR) | N = 17 | N = 13 |  |
| Age (years) | 56 ± 6 | 62 ± 4 | 0.01 |
| Gender (Females(%)) | 10 (59) | 6 (46) | 0.37 |
| Height (mt) | 1.68 ± 0.08 | 1.69 ± 0.11 | 0.80 |
| Weight (Kg) | 79.82 ± 23.03 | 72.51 ± 9.74 | 0.29 |
| SBP (mmHg) | 111 ± 9 | 134 ± 9 | < 0.001 |
| DBP (mmHg) | 70 ± 8 | 75 ± 9 | 0.10 |
| Creatinine (gr/dL) | 1.02 ± 0.21 | 1.01 ± 0.29 | 0.93 |
| GFR by Creatinine Clearance (mL/min) | 86.5 (79 - 100) | 88.73 (77.7 - 94.5) | 0.72 |
| Aldosterone (ng/dL) | 8.6 (3.9 - 15.3) | 6.9 (5.8 - 17.7) | 0.82 |
| PRA (ng/mL/hr) | 0.6 (0.16 - 1.6) | 0.6 (0.21 - 1.6) | 0.39 |
| cGMP_Plasma (pmol/mL) | 1.4 (1.1 - 2) | 1.3 (1.1 - 1.5) | 0.29 |
| cGMP_Urine (pmol/mL) | 408.6 (270.1 - 620) | 322.7 (259.3 - 495) | 0.54 |
| ANP (pg/mL) | 15.1 (2.5 - 27.9) | 2.8 (2 - 7.3) | 0.15 |
| NTproBNP (pg/mL) | 54 (32 - 156) | 32.2 (10 - 57.8) | 0.07 |
| LV wall Thickness (mm) | 9.33 ± 1.16 | 9.75 ± 0.59 | 0.31 |
| EF (%) | 64 ± 4 | 62 ± 6 | 0.49 |
| E wave velocity (m/sec) | 0.73 ± 0.16 | 0.69 ± 0.15 | 0.48 |
| A wave velocity (m/sec) | 0.62 ± 0.12 | 0.68 ± 0.14 | 0.26 |
| E/A ratio | 1.24 ± 0.42 | 1.03 ± 0.24 | 0.17 |
| E wave deceleration time (msec) | 192 ± 34 | 189 ± 16 | 0.75 |
| Left Atrial Volume Indexed (mL/m2) | 29 ± 9 | 27 ± 7 | 0.51 |
| Cardiac Index (L/m2/min) | 3.05 ± 0.52 | 2.92 ± 0.5 | 0.54 |
| E' velocity (m/sec) | 0.09 ± 0.02 | 0.09 ± 0.02 | 0.80 |
| E/E' ratio | 8.5 ± 2.9 | 7.7 ± 2.2 | 0.42 |
| Longitudinal sSR, Global Average (s/-1) | -1.41 ± 0.19 | -1.45 ± 0.2 | 0.64 |
| Longitudinal SR-E, Global Average (s/-1) | 1.73 ± 0.32 | 1.81 ± 0.39 | 0.62 |
| Longitudinal sS, Global Average (%) | -22.03 ± 2.03 | -22.56 ± 2.26 | 0.57 |
| Radial sSR, Average (s/-1) | 2.6 ± 1.08 | 2.25 ± 0.63 | 0.39 |
| Radial SR-E, Average (s/-1) | -2.82 ± 0.81 | -1.95 ± 0.73 | 0.02 |
| Radial sS, Average (%) | 52.44 ± 18.59 | 49.42 ± 13.93 | 0.68 |
| Circumferential sSR, Average (s/-1) | -1.93 ± 0.4 | -2.14 ± 0.6 | 0.33 |
| Circumferential SR-E, Average (s/-1) | 2.2 ± 0.51 | 2.29 ± 0.54 | 0.69 |
| Circumeferential sS, Average (%) | -21.96 ± 4.63 | -23.94 ± 6.05 | 0.40 |
| Left Ventricular Torsion (degree) | 27.61 ± 7.95 | 30.01 ± 14.83 | 0.71 |
| Torsion Rate (degree/sec) | 180.72 ± 63.38 | 185.74 ± 47.12 | 0.89 |
| Detorsion Rate (degree/sec) | 39.5 ± 180.4 | 93.38 ± 169.93 | 0.62 |
